# Chatbots and Diabetes: Is There Gender Bias?

**DOI:** 10.1177/23743735251380954

**Published:** 2025-09-24

**Authors:** Gloria Wu, Swara Tewari, Adrial Wong, Emily Chung, Ivan Chim, Brian Hoang, Nikki Mansubi, Adam Shams, Milan Del Buono, Sahana Srinivasan, Hrishi Paliath-Pathiyal, Obaid Khan

**Affiliations:** 1 Department of Ophthalmology, 12224 University of California, San Francisco School of Medicine, San Francisco, CA, USA; 2 Department of Biology, 118556 University of California, Santa Barbara, Isla Vista, CA, USA; 3 Department of Biology, 117238 University of California, Davis, Davis, CA, USA; 4 Department of Biology, 1846 Boston University, Boston, MA, USA; 5 Department of Biology, 8784 University of California, San Diego, La Jolla, CA, USA; 6 Department of Computer Science, 140245 University of California, Davis, Davis, CA, USA; 7 Department of Biology, 7161 San Jose State University, San Jose, CA, USA; 8 Department of Ophthalmology, 8785 University of California, San Francisco School of Medicine, San Francisco, CA, USA; 9 Department of Engineering, 12224 University of California, Berkeley, Berkeley, CA, USA; 10 Halmos College of Arts and Sciences, 2814 Nova Southeastern University, Fort Lauderdale, Florida, USA; 11 College of Osteopathic Medicine, 384448 California Health Sciences University, Clovis, CA, USA

**Keywords:** diabetic retinopathy, type 2 diabetes, artificial intelligence, patient education

## Abstract

This study evaluated 4 leading Large Language Models’ (LLMs) (ChatGPT-o1, DeepSeek-v3, Gemini 2.0 Flash, and Claude 3.7 Sonnet) responses to a question about Diabetic Retinopathy. **Methods:** The following questions were posed to the 4 LLMS: “I am a 52-year-old Caucasian [male/female] with out-of-control Type 2 Diabetes Mellitus, and I recently cannot read small print. What should I do?” We analyzed each response using Flesch-Kincaid Grade Level scoring and conducted a content analysis of the responses to evaluate for clinical terminology frequency, healthcare recommendations, and privacy considerations. **Results:** All platforms generated content at high school to college grade reading levels, exceeding recommended sixth-grade health literacy guidelines. DeepSeek incorporated more specialized clinical terminology and referenced specific diabetes guidelines not mentioned by ChatGPT, and exhibited greater gender discrepancy than the other 3 LLMs. **Conclusion:** While LLMs demonstrate promising capabilities for diabetes education, our results indicated that improvements in readability, gender bias mitigation, and risk of inappropriate output remain essential. Healthcare providers and physicians must review and monitor the answers before sharing with patients.

## Introduction

In 2021, 38.4 million Americans, or 11.6% of the American population, had diabetes mellitus, and it was reported to be the eighth leading cause of death in 2021.^1^ Despite the high incidence of diabetes in America, there are only 19,500 Certified Diabetes Care and Education Specialists, showing a significant gap in health education resources for Americans with uncontrolled diabetes.^2^

Access to high-quality diabetes education resources is critical as diabetic patients are at an increased risk of developing related health complications, such as diabetic retinopathy (DR), a leading cause of blindness.^3^ Anatomical changes to the center of the eye occur during both nonproliferative DR and proliferative DR, leading to diabetic macular edema.^4^

Patients often describe early signs of visual loss as loss of central letters in reading or report seeing a “smudge” when looking into the distance.^4^ These subtle changes are sine qua non for diabetic macular edema.^4^ These changes may be missed by optometrists and general internists but can be seen by advanced imaging tools such as optical coherence tomography.^5^

While all diabetic individuals are at risk of developing DR, those with low education and income status are more likely to have worse diabetes disease management outcomes, as lower health literacy is often a sequela of lower socioeconomic status.^6,7^

Given the limited accessibility of diabetes education resources, the lay public often turns to search engines for health-related queries. In fact, 7% of Google's one billion daily queries, or 77,000 searches per minute, are health-related.^8^ Large Language Models (LLMs) have emerged as an increasingly popular alternative.^9^ A major challenge with LLMs is their readability. According to US Census data, while 91.9% of Americans have completed high school,^10^ the average American reads at a seventh to eighth grade level. Additionally, 50% of Americans cannot understand a text written at the eighth-grade level.^11^

In this study, we explore how 4 LLMs—ChatGPT, DeepSeek, Claude.ai, and Gemini AI—respond to a simulated patient query related to diabetic vision loss. These 4 LLMs comprise the top 4, in terms of market share.^12^ ChatGPT, launched on November 30, 2022, is the oldest and most popular LLM, processing one million queries per day. ChatGPT uses a Generative Pre-Trained Transformer model of Machine Learning (MI) and reinforcement learning and has proprietary software developed by OpenAI (OpenAI, San Francisco).^13^

Claude.ai is unique among LLMs due to its “constitution” created by its software developers. This constitution serves as guardrails and guidelines for its AI and ML software, called Constitutional AI (CAI). After CAI, no additional human input is needed.^14^

Gemini, while not matching ChatGPT's query volume, leverages Google's vast search infrastructure, with 8.5 million total searches daily.^15^ Gemini utilizes PaLM2 and DeepMind technologies, with DeepMind being the ML algorithm combined with neural network architecture that famously defeated the Go Master in Seoul, Korea, in 2016.^16^

DeepSeek differs significantly from the other 3 LLMs in both foundational architecture and specialization. It focuses on code generation and technical problem-solving capabilities, using open source code, which ChatGPT, Claude.ai, and Gemini do not.^17^ DeepSeek's models are specifically trained on technical documentation, making it particularly adept at coding tasks, processes large amounts of code simultaneously and at a lower cost.^18^

We selected the American Academy of Ophthalmology (AAO) Preferred Practice Patterns (PPPs) for keyword evaluation of the LLM responses.^19^ PPPs are evidence-based guidelines developed by panels of ophthalmologists that provide standardized recommendations for diagnosing and treating various ophthalmic conditions. These guidelines are typically updated every 5 years to incorporate the latest scientific evidence and are designed to help ophthalmologists communicate effectively with patients about their care.^19^

Gender bias in LLMs has been documented in the literature.^20,21^ However, recent algorithmic improvements and prompt engineering advances have addressed many of these issues, making this an opportune time to reassess gender bias in contemporary LLM applications for healthcare.

## Methods

We used one query, varying by gender, across the 4 platforms: ChatGPT o1 (OpenAI, San Francisco), Claude 3.7 Sonnet (Anthropic, San Francisco), DeepSeek-v3 (Hangzhou Deeply Seeking Artificial Intelligence Basic Technology Research Co., Ltd, Hangzhou, China), Gemini 2.0 Flash (Alphabet, Inc, Mountain View). The study was conducted on March 31, 2025. We posed the following query:

“I am a 52-year-old Caucasian [male/female] with out-of-control Type 2 Diabetes Mellitus, and I recently cannot read small print. What should I do?”

The LLMs answer the queries based on the user prompt. Prompt engineering should be clear and show context, question, format, and examples.^22^ In creating our query, we used the specific age of 52 years old to both provide context and to denote menopause for women, as menopausal women have an increased prevalence of type 2 diabetes.^23^ The query also includes the complaint of “cannot read small print,” which implies decreased near vision and strongly suggests diabetic macular edema to ophthalmologists.^22^ To internal medicine physicians, the decreased near vision suggests diabetic retinopathy. This is another contextual cue for the LLM.^22^ We chose to keep all aspects consistent for both queries and varied only the gender of the user.

While gender varies across the 2 queries, the rest of the query was not changed as it contains “keywords” which are important to us as physicians who take care of diabetic patients and diabetic eye disease. This is a pilot study exploring gender bias and is not meant to be a comprehensive analysis of all LLMs and all possible medical queries.

We analyzed responses using the Flesch-Kincaid readability metric to assess text clarity and complexity.^24^ We computed the Flesch Reading Ease score, which quantifies readability with higher values indicating greater comprehensibility, and the Flesch-Kincaid Grade Level (FKGL), which determines the US school grade level required for understanding. These metrics enabled objective measurement and direct comparison of LLM language generation abilities.

The FKGL Formula assigns numerical scores representing the US grade level required for comprehension.^24^ This test is employed by the US Department of Education for evaluating reading levels of educational materials.

The formula results in a numerical value that represents the “mean number of years of education” generally required to understand the text.

FKGL  = 0.39 * (words/sentences) + 11.8 * (syllables/words)−15.59

Flesch-Kincaid Reading Ease = 0.39 × (words/sentences) + 11.8 × (syllables/words)−15.59

Keywords were selected by one of the physician authors who is an ophthalmologist. We chose to use the AAO Preferred Practice Guidelines for diabetic retinopathy for keyword selection.^19^ The AAO PPPs are designed to identify characteristics and components of quality eye care, guiding clinical practice.^19^ The PPPs are developed by a panel of Board Certified Ophthalmologists with expertise in the guideline topic, best available scientific data as published by peer-reviewed journals. A draft of the PPP is then reviewed by the entire PPP committee of ophthalmologists, the Committee of Secretaries, the Board of Trustees, the Council, subspecialty societies, national medical societies, and relevant patient organizations.^19^ Keyword identification was manually performed by the authors.

We used Cosine Similarity Scores to compare the male and female responses across all 4 LLMs. Cosine Similarity Scores are numerical values derived through vector analysis, denoting the level of similarity between 2 texts based on the presence of similar or identical words and phrases. Higher scores are indicative of greater similarity in the 2 texts.

To further evaluate for gender bias, we used ChatGPT-4.5 to compare the responses from the 4 LLMs. We chose ChatGPT-4.5 for this analysis as it is the newest version of the most widely used LLM and has advanced analytic capabilities.^25^ We uploaded the query responses from all 4 LLMs to ChatGPT-4.5 and added the following query:

“Compare the four LLMs’ responses for gender bias.”

All LLM answers were evaluated without additional prompting or clarification. Responses were analyzed as delivered to mirror realistic patient usage. To prevent bias, each response was assessed independently by 2 reviewers, blinded to the model that generated the text.

## Results

Table 1 demonstrates how all LLMs produced responses written at reading levels ranging from tenth grade to college level, exceeding the recommended sixth-grade level for health literacy communication. FKGLs varied from 10.5 to 14.3, with Gemini producing the most accessible content (10.5 grade level for female prompt) and Claude.ai generating the most complex (14.3 for male prompt). Flesch-Kincaid Reading Ease scores ranged from 31.7 to 44.5, indicating relatively difficult readability across all platforms.

**Table 1.** Reading Levels of Responses.

	ChatGPT female	ChatGPT male	Claude female	Claude male	DeepSeek female	DeepSeek male	Gemini female	Gemini male
Flesch-Kincaid Reading Ease	31.7	41.5	33.3	32.2	39.3	37	44.5	39.4
Flesch-Kincaid Grade Level	13.8	12.5	12.4	14.3	11.1	11.6	10.5	11.5

 Table 2 demonstrates how no significant difference was found in the length of the responses to the male and female queries (*p* = .844).

**Table 2.** Word Counts.

	ChatGPT o1	Claude 3.7 Sonnet	DeepSeek-V3	Gemini 2.0	Average	P-value
Male	359	180	413	360	328	0.844
Female	361	197	346	355	314.8	

While the *p*-value of .844 suggests no statistically significant difference between male and female performance across these AI models, based on algorithms and training datasets. In a way, statistical analysis is a challenge in this setting. Word counts are an imperfect way to judge a response. We use it because it has been used in prompt engineering.^22^ Thus, the comparison of LLM answers using word count is limited.

 Table 3 shows how keyword analysis reveals overlap and divergence among the 4 chatbots. “Eye exams” and “Blood sugar monitoring” were consistently included across genders and platforms. ChatGPT, Claude.ai, and Gemini each showed single-keyword gender variations: ChatGPT included “Endocrinologist” for females only, Claude.ai included “Diabetic Macular Edema” for females only, and Gemini included “Kidney” for males only.

**Table 3.** Keywords.

	Chatbots									
	ChatGPT o1		Claude 3.7 Sonnet		DeepSeek-V3		Gemini 2.0			
Keywords	Female	Male	Female	Male	Female	Male	Female	Male		Gender discrepancy
Eye exam	Yes	Yes	Yes	Yes	Yes	Yes	Yes	Yes	8/8	No
Eye MD	Yes	Yes	Yes	Yes	No	Yes	Yes	Yes	7/8	Yes
Urgency	Yes	Yes	Yes	Yes	No	Yes	Yes	Yes	7/8	Yes
Diabetic Mac Edema	No	No	Yes	No	No	No	No	No	1/8	Yes
Kidney	No	No	No	No	No	Yes	No	Yes	2/8	Yes
Foot	No	No	No	No	No	Yes	No	No	1/8	Yes
Exercise	Yes	Yes	No	No	Yes	Yes	Yes	Yes	6/8	No
Monitor blood sugar	Yes	Yes	Yes	Yes	Yes	Yes	Yes	Yes	8/8	No
Endocrinologist	Yes	No	Yes	Yes	Yes	Yes	Yes	Yes	7/8	Yes
Diabetes education	Yes	Yes	No	No	Yes	No	Yes	Yes	7/8	Yes
	7/10	6/10	6/10	5/10	5/10	8/10	7/10	8/10		

DeepSeek demonstrated the highest gender variation, mentioning “Eye MD,” “Urgency,” “Kidney,” and “Foot” for male responses but not female. Notably, the female query to DeepSeek yielded the fewest keywords. DeepSeek's inferior performance for female queries reveals concerning gender gaps in specialist referrals and urgency messaging.

## Cosine Similarity Score

The Cosine Similarity Scores comparing the male and female responses for ChatGPT o1, Claude 3.7 Sonnet, DeepSeek-V3, and Gemini 2.0, were, respectively: 0.789, 0.700, 0.821, and 0.864. The scores reveal that the Claude.ai male and female responses are most dissimilar.

 Table 4 shows how ChatGPT-4.5 evaluated the female responses as “empathetic” and tonally “warm” while male responses were described as more “clinical” in tone. ChatGPT-4.5 assigned scores to all of the LLMs, with higher scores indicating less gender bias. The scores it assigned ranged between 9 and 10, indicating low levels of gender bias for all 4 LLMs. ChatGPT-4.5 did not explain which parameters it used in its final scores, but in our human evaluation, we found there was a gender discrepancy for 7 of 10 keywords.

**Table 4.** ChatGPT-4.5 AI- Evaluation.

Chatbot	Female response characteristics	Male response characteristics	Gender bias observed	Score (/10)
Claude 3.7 Sonnet	Empathetic	Nearly identical	No	10
DeepSeek-v3	Empathetic	More concise	Minimal	9.5
Gemini 2.0 Flash	Warm emotional affirmations	Clinical	Tone difference	9
ChatGPT-o1	Empathetic	Clinical	Tone bias	8.5

## Discussion

All 4 of the LLMs differ in foundational architecture and specializations. ChatGPT, developed by OpenAI, prioritizes versatile conversational abilities across diverse domains. Its training emphasizes Reinforcement Learning from Human Feedback (RLHF) to align with human preferences and expectations in dialogue.^13^ ChatGPT's development path has concentrated on refining its ability to follow instructions precisely while maintaining safety guardrails, resulting in a more generalist approach to problem-solving compared to DeepSeek's technical specialization.^13^

Gemini, developed by Google, distinguishes itself through multimodal capabilities integrated at its core architecture rather than as additional features. It was designed from inception to process and reason across text, images, audio, and video simultaneously.^26^ Gemini's training incorporates Google's vast knowledge resources and emphasizes factual accuracy and scientific reasoning.^27^ Gemini uses deep learning and transformer-based software for its LLM. It is unclear if imaging data analysis is used in Gemini's answers.^26^

Some authors note that ChatGPT effectively equalizes information access while using a conversational voice.^28^ ChatGPT demonstrates limitations in verifying medical facts, a critical consideration for clinicians relying on evidence-based practice.^29^ Gemini now has citations or references to NIH websites to demonstrate the veracity of its data searches.^29^

Claude.ai, created by Anthropic, differentiates itself through its Constitutional AI. Claude.ai's development centers around harmlessness, helpfulness, and honesty principles encoded into its training “constitution.” Its architecture excels at understanding nuanced instructions and producing thoughtful, detailed responses with particular strength in reasoning through complex ethical scenarios and generating creative content.^30^ Shetty et al^30^ compared ChatGPT to Gemini and Claude.ai and found Claude.ai to have the fewest biases.

All 4 LLMs demonstrated gender disparity, with DeepSeek showing the greatest bias. This disparity may reflect regional training data patterns, as China has higher male diabetes prevalence and lower social service utilization among female elderly.^31^ These demographic patterns in training data may be contributing to gender bias in DeepSeek's responses. It is currently unclear how this bias will impact patient understanding and decision making.

LLM adoption in healthcare is accelerating. Physician interactions with these systems contribute to ongoing AI training, as do the millions of ChatGPT queries received daily. Healthcare institutions are implementing LLMs to reduce physician burnout by streamlining medical record documentation and improving workflow efficiency. AI-mediated ambient dictation has already enhanced workflow for emergency department physicians and nurses across the United States.^32^

## Readability

All 4 LLMs produced text at tenth grade to college reading levels. However, 54% of the US public reads at a sixth-grade level,^11^ creating significant barriers to accessing LLM-provided information for most Americans.

LLMs bridge linguistic barriers as ChatGPT and Gemini offer 50 + languages for 4.5 billion people. Claude.ai offers major languages, though the exact number is unclear. DeepSeek's linguistic capabilities remain undefined.

LLM readability can be manipulated through targeted prompt engineering, which trains models to recognize context, questions, format, and examples.^33^ Users can add prompts like “please answer at a sixth grade reading level.” However, reducing reading levels may conceal important information. Maintaining vital content while lowering reading levels may require physician input.

## Other LLMs

We selected 4 LLMs based on distinct characteristics and market positioning. ChatGPT was chosen due to its widespread adoption (650 million monthly users); Gemini for its Google search integration (1 billion daily queries); Claude.ai for its Constitutional AI training approach^14^; and DeepSeek for its open-source framework and non-Western origin.

Microsoft's Copilot was excluded as it utilizes OpenAI technology. Cohere was omitted due to declining market share since 2022.^12^ Perplexity.com and Meta's platforms were not included due to smaller user bases compared to our selected models.

## Patient Safety and Ethics

Our study revealed factual accuracy in LLM responses but identified gender bias and lack of empathetic communication that patients expect from healthcare interactions. Current LLMs cannot replicate nuanced communication skills required for anxious patients and family members.^34^

The landscape is rapidly evolving with emotionally responsive AI development. Character.ai's conversational chatbots simulate emotional responses, introducing unprecedented risks, as demonstrated by a recent suicide case following Character.ai interactions.^35^ Such incidents underscore the critical need for robust safety protocols and regulatory oversight.

## Privacy and Security Risks

LLMs present opportunities and challenges for medical practice as patients increasingly utilize these accessible tools. However, public policy guidelines governing LLMs in health education remain absent, creating patient safety and information quality risks.^36^

LLMs trained on problematic data sources pose significant healthcare risks. Grok, trained on X (formerly Twitter), incorporates profanity and misinformation from user entries, potentially contaminating health education and threatening evidence-based medicine.^37,38^

While all LLMs require user consent for data collection, DeepSeek stores data on Chinese government-controlled servers. Consequently, both US and Australian governments have prohibited government employee use.^39^ For this reason, DeepSeek's clinical utility in the future will likely be limited in American healthcare.

## Clinical Practice

The top AI-mediated LLMs compete for user attention. Notably, Grok contains significant “bot” users disseminating misinformation and conspiracy theories.^37^

Healthcare providers must help patients navigate this space. Physician engagement with LLMs can improve training quality and accuracy of healthcare information. Stanford University and Mayo Clinic have partnered with OpenAI to work on an internal LLM for their own organizations.^40^ This indicates the future involves LLM giants partnering with hospitals to create LLMs with verifiable medical information following clinical guidelines.

## Future Research

LLM improvement requires diverse healthcare provider input during development to reduce bias and increase response validity. Training LLMs using approved clinical guidelines from medical associations would reduce healthcare information gaps.

All LLMs currently offer free trials to physicians, students, and academic faculty. Application Programming Interface (API) opportunities exist for healthcare input, with institutional IT departments providing implementation support for noncoding physicians.

## Conclusion

Our comparative analysis reveals that LLMs provide medical knowledge access within 20 s, creating profound implications for patient education. Each platform demonstrates unique strengths: DeepSeek offers computational efficiency, ChatGPT provides conversational versatility, Gemini delivers factual accuracy with citations, and Claude.ai employs Constitutional AI approaches. In our study, the LLMs exceed recommended readability levels and exhibit some gender disparities requiring attention.

Our findings indicate that using multiple LLMs yields superior results compared to single-platform reliance. We recommend patients and clinicians utilize at least 2 LLMs—preferably ChatGPT and Gemini—to cross-reference responses while physicians monitor integration to optimize patient education in clinical settings.
